# Snail plots are badges of genome assembly quality

**DOI:** 10.1093/g3journal/jkag074

**Published:** 2026-04-06

**Authors:** Richard Challis, Mark Blaxter

**Affiliations:** Tree of Life, Wellcome Sanger Institute, Hinxton, Cambridge CB10 1SA, United Kingdom; Tree of Life, Wellcome Sanger Institute, Hinxton, Cambridge CB10 1SA, United Kingdom

**Keywords:** data visualization, genome assembly, quality assessment, assembly metrics

## Abstract

Assembly quality is frequently assessed using independent measures of assembly span, contiguity, sequence composition, and completeness. Among contiguity metrics, contig, and scaffold N50 have perhaps gained the most traction, despite well-known limitations. Several authors have suggested considering the complete N*x* curve rather than just the N50 value, but while using N90 values or considering the area under the N*x* curve with auN statistics provide more complete measures of contiguity, they share the limitation of being unsuited to direct comparison across a range of genome sizes. We introduced snail plots to provide a genome-size-independent way to summarize a range of commonly used assembly metrics. Here, we demonstrate that easily learnt visual differences between snail plots allow simultaneous consideration of metrics across several key areas of assembly quality to rapidly identify high- and low-quality assemblies. We show that prominent features in snail plots of high-quality assemblies effectively highlight N50, N90, and auN contiguity statistics. As the presentation is scaled to the longest scaffold, we also show that plots can be compared effectively across a wide range of taxa and assembly sizes. We use the core features of a snail plot to derive a proportional measure of assembly quality based on auN, adjusted for non-ATGC bases and scaled to the length of the longest scaffold. We show that this “snail score” value corresponds closely to a qualitative assessment of overall assembly quality from visual interpretation of a snail plot and supports corrections for expected genome size.

## Teaser

Measures of genome assembly quality are used to inform downstream uses of genomic data. Many common metrics, however, focus on a single aspect of assembly quality and cannot be directly compared across divergent genome sizes. Challis and Blaxter formally describe snail plots as rich visualisations that represent a suite of these metrics in genome-size independent “badges” of assembly quality. The proportions of key plot features can be interpreted as signatures of high and low assembly quality. The authors use their observations of snail plot features to derive a genome-size independent snail score metric that corresponds closely to these assembly quality signatures.

## Introduction

Genome assembly is the process of reconstructing the DNA sequence of an organism from available sequencing data. Higher quality assemblies are those that provide a more accurate representation of the true DNA sequence. This accuracy is typically assessed using measures of sequence contiguity such as scaffold N50 or NG50 lengths, sequence base composition such as GC proportion, error rate, and recovery of expected marker genes, including BUSCO (Simão et al. 2015) sets.

While scaffold N50 (the length of the longest scaffold for which at least half of the assembly is in scaffolds of that size or greater) is among the most widely used metrics, it has known limitations. It may be biased upward by excluding shorter scaffolds that are part of the genome so the scaffold NG50 length (Earl et al. 2011) uses the known or expected genome size in place of the assembly span. It may also be biased upward by the misassembly of large scaffolds and does not reflect increases in contiguity for scaffolds below the N50 length. Several authors have addressed these last points by considering the wider family of N*x*/NG*x* statistics (where *x* is the percentage of the assembly/genome size) or the full N*x*/NG*x* curves for a set of assemblies (Bradnam et al. 2013) and defining a metric based on the sum of squared scaffold lengths divided by the sum of scaffold lengths as E-size (the expected size of a scaffold that would contain a gene chosen at random; Salzberg et al. 2012) or auN/auNG (the area under the N*x*/NG*x* curve; Li 2020). A further limitation is that this family of metrics is limited to the comparison of assemblies for the same taxon. Despite being commonly reported as a measure of assembly quality, they are dependent on genome size and on chromosome size distributions and cannot be directly compared across assemblies of taxa with different-sized genomes (Miller et al. 2010).

Full assessment of assembly quality requires consideration of a combination of these metrics, and several tools are available to perform quality assessments, often incorporating additional data from a reference assembly or mapped reads to allow assessments of correctness, e.g. QUAST (Gurevich et al. 2013), LASER (Khiste and Ilie 2015), Merqury (Rhie et al. 2020), WebQUAST (Mikheenko et al. 2023), and Genome Evaluation Pipeline (Sullivan et al. 2025). Such tools include tabular and graphical outputs, but interpreting the core statistics requires adjustment of expectations according to the overall expected genome size and chromosome count. Comparing tabulated results across assemblies also implies a focus on a subset of metrics and brings issues of inconsistent formatting when comparing across different source publications.

Snail plots were conceived as an assembly “badge” to provide a consistent way to convey a suite of these metrics for any assembly. Relative to tables of core metrics, snail plots convey a greater density of information, with a high “data-to-ink” ratio (Tufte 2001), making it easier to assess core aspects of assembly quality at a glance, use consistent formatting to reduce the cognitive load of comparing data from different sources, and are scale-independent; hence, expectations do not need to be adjusted based on the size of the genome under consideration. Snail plots also convey the distribution of scaffold lengths and provide similar insight to metrics such as auN/auNG. These distributions can highlight features of assemblies that may be missed by other metrics, such as a high number of very short scaffolds.

Snail plots were first introduced in the LepBase genome browser (Challis et al. 2016), for which they were implemented as a set of standalone scripts (Challis 2017). They have since been reimplemented as a core component of BlobToolKit (Challis et al. 2020), with snail plots for over 10,000 assemblies available on the public viewer at blobtoolkit.genomehubs.org/view. Snail plot assembly badges also appear as main figures in numerous genome assembly papers, including over 1,600 genome notes from the Darwin Tree of Life project (Mead et al. 2020; Aunin et al. 2021) and are presented in a modified form on WormBase ParaSite (Howe et al. 2017).

Here, we present a full formal description of snail plots and highlight updates to the plots and plot generation workflow since the study of Challis et al. (2020). We describe the most recent implementation of snail plots and provide best practice guidance for configuring, presenting, and interpreting the plots both as an assembly quality inference tool and to offer insight into high-level assembly organization. We describe new functionality to control the scaling and numerical data presentation, and the implementation as a component of the fully command-line–based BlobTK tool for generating snail plots from local or remote files without requiring the browser-based visualization of BlobToolKit. We also suggest a new metric based on auN that provides a single value for assembly quality that can be compared across assemblies of different sizes, supports corrections for expected genome size, and correlates closely with visual indicators of assembly quality observed in snail plots.

## Methods

### Calculating summary statistics

Snail plots represent a graphical summary of assembly statistics derived from the primary assembly sequence (FASTA) file and a BUSCO full table (TSV) output file, for which any relevant BUSCO lineage may be used, but convention is to use the most specific lineage available. These files may be read directly to generate a snail plot using the BlobTk command line tool or from the processed data in a BlobDir dataset to allow full integration with the BlobToolKit pipeline (see *Implementation*, below). In either approach, the data are processed to extract length, base composition data (counts of AT, GC, and N bases), together with identities and counts of complete, duplicated, and fragmented BUSCO genes for each sequence. Values are stored associated with individual sequences to allow for an optional filtering step based on sequence properties, described in *Plot options*, below.

Total assembly span is calculated as the sum of filtered sequence lengths, while the longest scaffold length and scaffold N50 and N90 lengths are obtained from the size-sorted list of scaffold lengths. N50 and N90 values are calculated as the length of sequence for which the cumulative sum of lengths is greater than or equal to 50% and 90% of the total assembly span, respectively. BUSCO proportions for each status (complete, fragmented, duplicated, and missing) are calculated from the full list of BUSCO genes across all included sequences.

Scaffold lengths, scaffold counts, and sequence base composition proportions are calculated for equal-sized bins across the assembly span based on the size-sorted scaffold list. The default presentation uses 1,000 bins; hence, each bin represents 0.1% of the total assembly span. Values for each bin are a summary of individual values for all scaffolds that overlap that bin, such that base composition values are the mean, minimum, and maximum values across all scaffolds that overlap a bin, the scaffold length for a bin is the length of the longest scaffold that overlaps the bin, and the cumulative scaffold count includes all scaffolds that overlap that and all previous bins.

### Snail plot composition

**Fig. 1.**
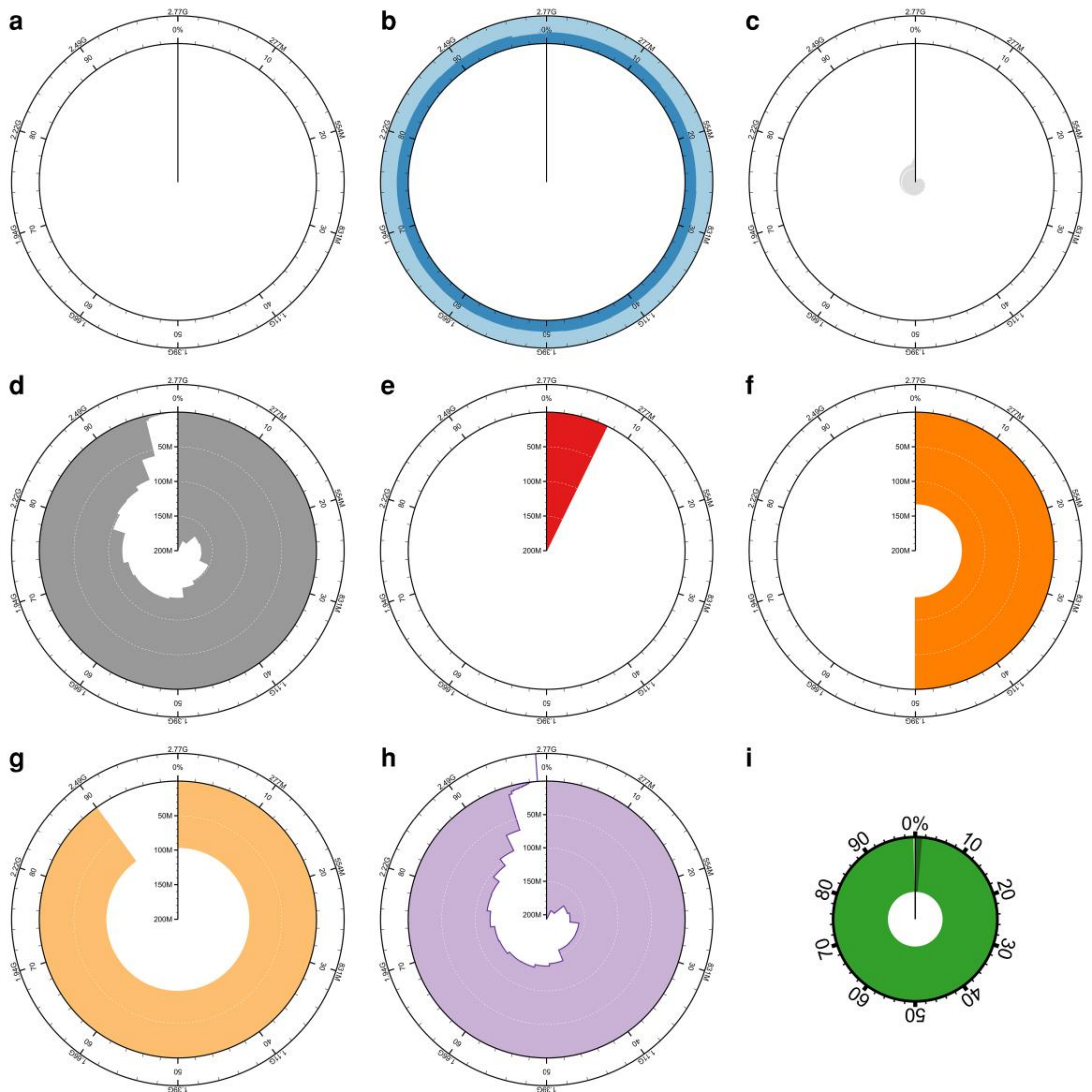
Snail plot features taken from a plot of *Mus musculus* assembly GCA_949316315.1. a) Circumferential axis showing cumulative and percentage span, default settings divide this into 1,000 bins of 0.1% assembly span each. b) Base composition track highlighting GC and AT proportion and showing proportion of Ns if present. c) Central light gray shading showing log-scaled cumulative count. d) Dark gray shading showing size-sorted scaffold lengths in each bin. e) Red shading highlights the proportional size of the longest scaffold in the assembly. f) Dark orange shading highlights the N50 scaffold length. g) Light orange shading highlights the N90 scaffold length. h) Optional purple line and shading representing a reference assembly scaffold length distribution to allow direct comparison, and (i) an inset plot show assembly BUSCO complete, duplicated, fragmented, and missing percentages. The full snail plot for this assembly is shown in Fig. 2a, and the reference assembly overlay from assembly GCA_964188535.1 is included in Fig. 7a.

Snail plots layer a suite of assembly metrics into a dense visual representation of assembly quality. The core plot components of a snail plot for a chromosomal assembly of the house mouse *Mus musculus* (GCA_949316315.1), sequenced for the Darwin Tree of Life project (Darwin Tree of Life Project Consortium 2022), are dissected in Fig. 1. The base snail plot was generated using the command:

blobtk snail \

-d https://blobtoolkit.genomehubs.org/api/v1/dataset/id/GCA_949316315.1

Most graphical information is presented in a circular plot, where the circumferential axis is labeled with cumulative span and percentage of the total assembly (Fig. 1a). Data presented along this axis are drawn for the equal-sized bins representing scaffolds from the longest to the shortest clockwise around the plot.

The outer portion of the plot contains a base composition track with dark and light blue shading representing the GC and AT proportions, respectively (Fig. 1b). If scaffolds in a bin contain Ns, the proportion of Ns is represented by white shading at the edges of this track. Variation in GC content across multiple scaffolds in a bin is represented by lighter and darker shading to show the range of values about the mean.

At the center of the plot, a region of light gray shading indicates the cumulative scaffold count (Fig. 1c). The count is always shown on a log-scaled axis starting at a value of 1 closest to the center of the plot with solid white gridlines shown at order-of-magnitude intervals from 10 upward.

The lengths of the size-sorted scaffolds are shown in dark gray shading from the circumference to the center of the plot (Fig. 1d). The radial axis is scaled to the length of the longest contig in the assembly, and where multiple scaffolds are present in a bin, the shading represents the length of the shortest scaffold in that bin. Dashed white lines are shown at major gridlines to allow tracing scaffold length axis values around the plot.

The longest scaffold is highlighted with red shading (Fig. 1e). For assemblies with multiple scaffolds in the first bin, no red shading will be present, although the radial axis still represents the longest scaffold in the assembly. Dark and light orange shading represent the N50 and N90 scaffold lengths (Fig. 1f and 1g), respectively. These shaded areas are always present; however, they may not be visible for assemblies with low contiguity where the longest scaffold is orders of magnitude longer than the N50 and/or N90 value.

An optional reference assembly scaffold length overlay may be displayed using a purple line and shading (Fig. 1h). When shown, the shading is displayed below the main scaffold length distribution, while the line is displayed above, so that both the target and reference distributions remain fully visible for comparison. If the reference assembly span is shorter than the target assembly, the purple line is continued across the base composition track to provide a clear marker of the end of the reference scaffold distribution.

Outside the main plot area, a smaller circular plot represents the BUSCO completeness of the assembly (Fig. 1i). The shaded region represents the percentage of complete BUSCOs in the assembly, darker shading shows the percentage of duplicated BUSCOs and lighter shading shows the percentage fragmented.

### Interpreting key features

**Fig. 2.**
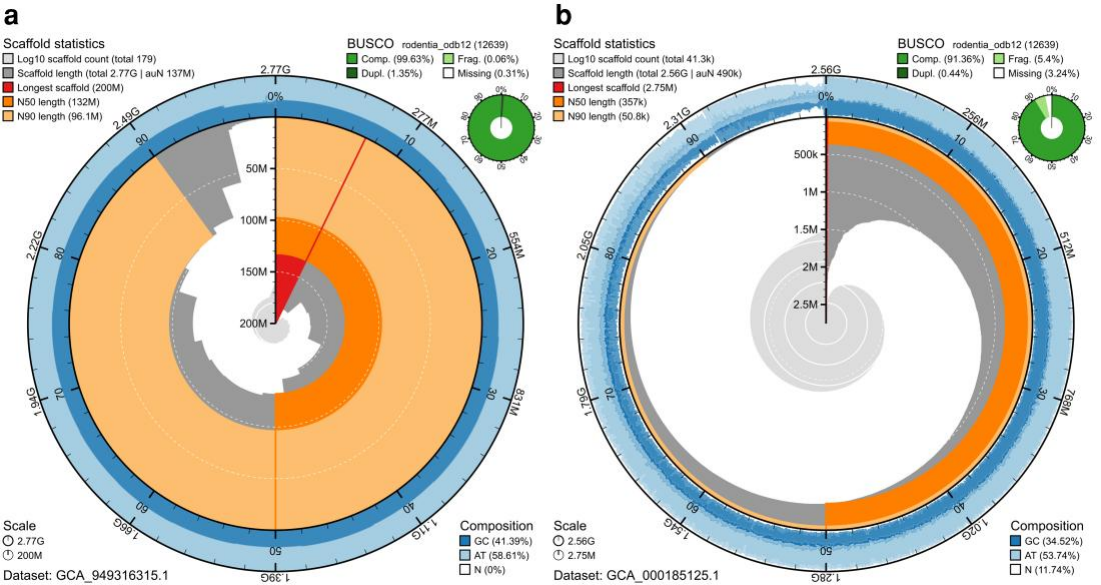
Snail plots for 2 assemblies of the house mouse, *Mus musculus*, highlight the visual contrast between (a) high-contiguity assembly GCA_949316315.1 and (b) low-contiguity assembly GCA_000185125.1.

The full snail plot for the chromosomal assembly GCA_949316315.1 is presented alongside a lower contiguity scaffold assembly (GCA_000185125.1) (Gnerre et al. 2011) in Fig. 2 to place the features described above in the context of assembly comparison. The snail plot for assembly GCA_000185125.1 was generated with the command:

blobtk snail \

-d https://blobtoolkit.genomehubs.org/api/v1/dataset/id/GCA_000185125.1

Table 1 presents a detailed side-by-side comparison of individual plot features for the 2 assemblies. Overall, the scaffold length curve occupies a much larger proportion of the plot area for assembly GCA_949316315.1 and has a stepped profile compared with the smooth curve for assembly GCA_000185125.1. The longest scaffold and N90 overlays are both more prominent for assembly GCA_949316315.1; the N50 overlay extends further toward the center of the plot, and its prominence is only reduced by the extent of the N90 overlay. Around the outside of the plot, assembly GCA_949316315.1 has a more consistent GC proportion compared to assembly GCA_000185125.1, which also has a higher proportion of whitespace on the composition track, indicating a higher proportion of N bases in the assembly. Assembly GCA_949316315.1 also has higher BUSCO completeness, assessed using the BUSCO 6.0.0 with the rodentia_odb12 set.

**Table 1.** Pairwise comparison of snail plot features for *Mus musculus* assemblies GCA_949316315.1 and GCA_000185125.1 as shown in Fig. 2a and 2b, respectively.

Attribute	GCA_949316315.1	GCA_000185125.1	Presentation
BlobToolKit Assembly ID	mMusMuc_1	AEKR01	Bottom left legend
Longest scaffold	Prominent; ∼200 Mb; ∼7% of assembly span	Barely visible; < 3 Mb	Red overlay
Scaffold N50	>100 Mb; close to longest scaffold length	∼400 kb; much shorter than longest scaffold length	Dark orange overlay
Scaffold N90	Prominent; 90–100 Mb; high relative to N50	<<100 kb; low prominence relative to N50	Light orange overlay
Scaffold length curve	Mostly covered by overlays; extends close to center of plot; stepped appearance; ∼4% of assembly has relatively short scaffolds (<10 Mb)	Prominent areas not covered by overlays; smooth spiral away from center of plot; no obvious break between long and short scaffolds	Dark gray shading
Scaffold count curve	Very small area shaded; only one prominent gridline so most of assembly in <100 scaffolds	Prominent; large area shaded with 3–4 prominent gridlines so highly fragmented assembly with >10,000 scaffolds	Light gray shading
Sequence composition	Consistent mean GC proportion throughout; slightly lower GC in shortest scaffolds; no visible white areas so very low proportion of Ns	Highly variable GC proportion; variation greater as scaffold length decreases; Prominent white areas all around track so high proportion of Ns throughout the assembly	Dark/light blue shading
BUSCO completeness	>99% complete; very low fragmented proportion; ∼1.4% duplicated	<92% complete; ∼5% fragmented; very low duplicated proportion	Green shading in top right inset

Many of these key differences are also apparent from inspection of the set of numerical values in the plot legends that would commonly be presented in a table. The chromosomal assembly GCA_949316315.1 has the longest scaffold, N50, and N90 lengths that are all orders of magnitude greater than the equivalent values for assembly GCA_000185125.1. There are also large differences in the number of scaffolds and the percentage of missing data, with the ∼41.3k scaffolds of assembly scaffolds of assembly GCA_000185125.1 having an average of 11.89% N bases compared to 0% N bases for the 179 scaffolds of assembly GCA_949316315.1.

### Implementation

Interactive snail plots have been available as part of the BlobToolKit Viewer (Challis et al. 2020). Snail plots may now be generated on the command line using the BlobTK subcommands blobtk plot --view snail and blobtk snail as part of a suite of tools that provide high-performance functions for processes commonly used in BlobToolKit and other GenomeHubs tools.

BlobTK is implemented in Rust for optimal performance when processing assemblies with their associated reads and read alignments. While these considerations are less directly relevant to the blobtk plot and blobtk snail commands, using the same codebase allows for efficient processing of JSON BlobDir files via serde (https://github.com/serde-rs/serde) and serde_json (https://github.com/serde-rs/json) and SVG generation with resvg (https://github.com/linebender/resvg). The command-line interface is implemented with clap (https://github.com/clap-rs/clap), and Python bindings to blobtk plot are implemented with maturin (https://github.com/PyO3/maturin) to allow the plotting functionality to be imported into Python code.

The command blobtk plot --view snail, may be used to generate a plot from an existing BlobDir dataset, either available as a local directory or remotely hosted via the BlobToolKit API. This is the standard BlobToolKit data format (Challis et al. 2020) https://pipelines.tol.sanger.ac.uk/blobtoolkit (Challis et al. 2020) https://blobtoolkit.genomehubs.org/view (Challis et al. 2020). The data summarized in snail plots are contained in the meta, gc, length, n, ncount, and (optional)_busco files. This represents a minimal BlobDir dataset; hence, this command is compatible with all valid BlobDirs generated by any version of BlobToolKit.

The command blobtk snail is a wrapper around the plot subcommand that adds support for direct import from any uncompressed or gzip compressed assembly FASTA file and (optional) BUSCO full table TSV file available on the local filesystem or accessible via ssh, ftp, or http. If FASTA/BUSCO files are provided directly, they are converted to a named or temporary minimal BlobDir before being processed identically to the plot command. The use of an intermediate BlobDir allows for the reuse of summary statistic calculations and filtering routines shared by other plot types. The BUSCO full table parser is compatible with output files from BUSCO versions 4, 5 (Manni et al. 2021), and 6 (Tegenfeldt et al. 2025) using odb10 or odb12 lineage datasets.

### Software availability and installation

Precompiled BlobTk binaries associated with each release are available for Linux and macOS and may be downloaded directly from the BlobTK GitHub repository releases page at https://github.com/genomehubs/blobtk/releases. The blobtk plot subcommand was introduced in version 0.3.0, and the full feature set described here is available in the 0.8.0 release, which is also the first release to include the blobtk snail subcommand. Releases are archived in Zenodo under the DOI 10.5281/zenodo.10560870.

BlobTk may be installed using the conda package manager using the command conda install tolkit::blobtk. The conda package includes both the BlobTK command line and Python module implementations. Python modules for BlobTk version 0.8.0 are compatible with Python versions 3.9-3.14 and may also be installed using pip with the command pip install blobtk.

A BlobTk container image is also available on Docker Hub and may be pulled using the docker command docker pull genomehubs/blobtk:latest or the singularity command singularity pull blobtk.sif docker://genomehubs/blobtk:latest. Tagged images are also available for each release, e.g. genomehubs/blobtk:0.8.0.

For users of BlobToolKit, BlobTk version 0.8.0 is also included in the genomehubs/blobtoolkit:4.5.3 Docker image.

### Making a snail plot

#### Plot generation

BlobTk supports snail plot generation from local and remote files.

All examples in this study use hosted BlobDir datasets available on the public BlobToolKit viewer and can be generated by specifying the corresponding API URL, e.g. for the plot of assembly GCA_949316315.1 in Fig. 1:

blobtk snail

-d https://blobtoolkit.genomehubs.org/api/v1/dataset/id/GCA_949316315.1

To generate a plot from a BlobDir dataset on a local filesystem, instead use the local directory path:

blobtk snail \

-d /path/to/local/blobdir

To generate a plot directly from a local or remote FASTA and (optional) BUSCO full table files, use the --fasta and --busco flags with local file paths or remote file URLs:

blobtk snail \

--fasta /path/to/local/assembly.fa

--busco http://example.com/path/to/local/full_table.tsv

When called in this way, BlobTk generates a temporary intermediate BlobDir directory. To store this intermediate BlobDir, include a BlobDir directory path in the above command (i.e. -d/path/to/local/blobdir). The intermediate BlobDir will then be written to the specified directory and will be available for use in subsequent commands in place of specifying the original FASTA and BUSCO paths directly. Alternatively, a BlobDir may be created using BlobToolKit either by running the full pipeline or with a minimal command of the form:

blobtools create \

--fasta /path/to/input/assembly.fasta

--busco /path/to/input/full_table.tsv \

/path/to/local/BlobDir

#### Plot options

Snail plots are intended to serve as assembly badges; while there is scope for some customization, the range of options is limited to ensure visual consistency of core plot features. Table 2 shows the available snail plot customization options in BlobTK version 0.8.0, including a subset of options that are only available via the blobtk snail wrapper command. Further documentation on the plotting options is available via the blobtk wiki pages at https://github.com/genomehubs/blobtk/wiki/blobtk-plot and https://github.com/genomehubs/blobtk/wiki/blobtk-snail.

**Table 2.** Snail plot customization options for use with the in blobtk plot –view snail and blobtk snail commands (version 0.8.0).

Option	Description	Default value
-o, --output	Output filename. Use .png/.svg suffix to render snail plot or .json/.yaml suffix to print all plot statistics to file	output.svg
-f, --filter	Apply a filter to exclude scaffolds based on any variable in the BlobDir dataset	
-s, --segments	Segment count	1000
--max-span	Maximum value on the circumferential axis	assembly span
--max-scaffold	Maximum value on the radial axis	longest scaffold length
--reference	Reference BlobDir or FASTA file	
--scale-function	Scale function for the radial axis (linear, sqrt or log)	linear
--show-score	Flag to show the snail score	false
--score-only^a^	Report snail score only to terminal	
--score-json^a^	Report snail score as JSON to terminal	
--score-type	Score variant to display with –show-score (base, g, gs, ag, ags)	base
--badge	Flag to show snail plot as an assembly badge with no legend or text	false
--significant-digits	Significant digits to use when rounding numbers for display	3
--decimal-precision	Number of decimal places to show when rounding percentages for display	2
--rounding	Rounding strategy (round, down or up)	round
--show-numbers	Flag to show absolute values instead of percentages	false
--busco-numbers	Flag to show absolute values instead of percentages for BUSCO counts only	false
--assembly-name^a^	Display name to use for the source assembly	
--reference-name^a^	Display name to use for the reference assembly	

^a^Option only available via the blobtk snail command.

Setting the --output filename sets the image format according to the filename suffix (png or svg) or allows for printing the processed plot data to a json or yaml file.

The --filter option restricts the list of scaffolds included in the plot. This is intended to be used in conjunction with the interactive BlobToolKit viewer on datasets processed using the full BlobToolKit pipeline, but it can also be used to see the impact of, for example, excluding contigs of less than 1 kb. The syntax for filtering is {field name}:{filter type}=value, where the primary filter types are min and max for numeric values and keys or inv for comma-separated lists of keyword values to exclude or include. The 1 kb length filter would be specified as --filter length:min=1,000. For full compatibility with BlobToolKit, a variant of this syntax using a—separator is also supported, i.e. --filter length--Min=1,000.

Snail plots are visually circular, but this is implemented as a polygon with a default of 1,000 sides. Within each segment, the GC and N values are summarized across all scaffolds in that bin. Reducing --segments to 100 increases the bin size for these summary statistics, which can reduce the noise in the GC track for portions of the assembly with large numbers of short contigs. Smaller values could be used to produce stylized plots at the expense of accuracy in representing proportional scaffold lengths.

Plots are intended to be scale-independent to allow assessment of assemblies regardless of the total assembly span or longest scaffold length. When comparing assemblies directly, the --max-span and --max-scaffold lengths can be set manually to produce plots with the same circumferential and radial ranges.

Plots can also be scaled to a reference assembly using the --reference option to specify the path to a BlobDir dataset or an assembly FASTA file. If a reference assembly is specified, the scaffold length distribution for the reference is overlaid on the snail plot using a purple line and fill. The --max-span, --max-scaffold, and --reference options interact such that the plot is scaled to the largest value across any of these options that is specified and that of the target assembly.

Snail plots were introduced before chromosome-level assemblies became commonplace; hence, scaffold lengths in versions prior to 0.8.0 were square-root scaled by default to increase visibility of trends in sequence length among the shortest scaffolds in the assembly. In assemblies with low contiguity, these short scaffolds can represent a high proportion of the data; however, for chromosomal assemblies, this emphasis on the shorter scaffolds reduces the resolution of differences in chromosome lengths. To address this, the --scale-function default has been changed to linear as of version 0.8.0.

A set of options controls the display and calculation of the auN-based snail score (see *Defining a snail score summary statistic*, below). The --show-score option adds the snail score directly to the plot, while --score-only and --score-json options can be used to write the snail score directly to the terminal. A further --score-type option can be used to apply genome and scaffold size–based adjustments to the snail score (see *Correcting for expected genome size*, below).

Snail plots are intended to convey more nuanced information than individual assembly metrics, such as N50, can provide without reference to the underlying values. The --badge option fully realizes the potential of snail plots to act as assembly badges by removing all legends and numerical annotation from the plot. To account for the fact that visual cues for the plot scale may not be visible when presented as a badge, the --badge option enforces --scale-function linear.

The remaining options (Table 2) all concern the display and formatting of the plot legend and axis tick labels. These allow control of the rounding behavior and significant digits/precision separately for absolute values and percentages and displaying absolute values instead of percentages. These options do not affect the visual consistency of the core plot features but allow for avoiding issues with rounding to the same value when comparing assemblies with similar BUSCO completeness in lineages with large numbers of BUSCO genes.

### Comparing scale options

The low-contiguity *Mus musculus* assembly GCA_000185125.1, shown in Fig. 2b, was replotted using alternative scaling to show the effect of the default and alternative scale options on the plot appearance.

The default linear scale function (as presented in Fig. 2) provides an accurate representation of the full scaffold length distribution and allows differences between the lengths of the longest scaffolds to be more easily seen, and is the most appropriate for high-contiguity assemblies. Versions of BlobTk prior to 0.8.0 used a square-root scale function to emphasize differences between shorter scaffold lengths in the more highly fragmented assemblies that were more typical when snail plots were first introduced. Plotting with a square-root scale is still available as an option; hence, variants of the snail plot for assembly GCA_000185125.1 were produced with a square-root scale to allow direct comparison using the command:

blobtk snail \

-d https://blobtoolkit.genomehubs.org/api/v1/dataset/id/GCA_000185125.1 \

--scale-function sqrt

By default, plots are scaled to the total span and maximum scaffold lengths of the assembly. To show the effect of this relative to scaling to an expected genome size and maximum scaffold length, 2 variants of the square-root-scaled assembly were produced, scaled to the assembly span of the high-contiguity assembly GCA_949316315.1 using --max-span 2770968735 and scaled to both the assembly pan and longest scaffold length of assembly GCA_949316315.1 by adding the option --max-scaffold 200127270.

### Correlation with standard metrics

Most assembly metrics are tied to the assembly span or genome size of the organism being sequenced. To demonstrate the scale independence of snail plots relative to the commonly used scaffold and contig N50 metrics, a set of assemblies at the scaffold, chromosome, or complete genome level was obtained from the 2025.04.21 archive release of Genomes on a Tree (GoaT) (Challis et al. 2023). Assemblies were grouped into bins by order of magnitude of scaffold and contig N50 values, and a single representative assembly was chosen pseudo-randomly from each bin using the Python random module with a seed of 1,031. The corresponding BlobToolKit ID was identified for each assembly and used to generate a snail plot using a blobtk snail command, with output files saved in SVG and YAML formats. An example command for the high-contiguity *Mus musculus* assembly GCA_949316315.1, which is included in this set is:

blobtk snail \

-d https://blobtoolkit.genomehubs.org/api/v1/dataset/id/GCA_949316315.1 \

--badge \

-o GCA_964340765.1_badge.svg

-o GCA_964340765.1_badge.yaml

SVG files for each plot were combined into a single file to generate a grid of snail plots on axes of increasing contig and scaffold N50, with 1 plot per occupied bin. This process was automated using the docs/snail-plots/figure 4.py script in the BlobTk repository.

### Taxonomic distribution of example assemblies

In selecting example assemblies across a wide range of traditional assembly metrics, the set of assemblies used for comparison with standard metrics included representatives from the 3 major eukaryotic kingdoms. To assess the relative influence of taxonomy, a taxonomic tree view of the included assemblies was generated using the 2025.04.21 archival release of GoaT. The tree was colored by taxonomic kingdom with bars to indicate assembly span using the URL https://goat.genomehubs.org/search?query=assembly_id=GCA_900322205.1%2CGCA_003016195.1%2CGCA_001632505.1%2CGCA_000261425.2%2CGCA_002222395.1%2CGCA_020883555.1%2CGCA_001661245.1%2CGCA_000204055.1%2CGCA_964340765.1%2CGCA_013467465.1%2CGCA_014337955.1%2CGCA_018257905.1%2CGCA_964340405.1%2CGCA_000001215.4%2CGCA_003033685.1%2CGCA_013339765.2%2CGCA_963691655.1%2CGCA_949316315.1%2CGCA_019009955.1%2CGCA_964205295.1%2CGCA_963693085.1&result=assembly&taxonomy=ncbi&report=tree&collapseMonotypic=true&treeStyle=ring&y=assembly_span&cat=kingdom&hideSourceColors=true. The tree was manually edited to ensure all GCA accession labels were printed in full.

### Defining a snail score summary statistic

The area under the N*x* curve, auN (Li 2020), or E-size (Salzberg et al. 2012) was calculated by dividing the sum of squared scaffold lengths by the sum of scaffold lengths (or assembly span). By this formula, the value of auN will always be less than or equal to the length of the longest scaffold, and in practice, it can only be equal to the longest scaffold length if the assembly has a single scaffold or if all scaffolds are of equal length. As such, a relative measure of auN can be derived by dividing auN by the longest scaffold length to give a proportional value ≤ 1 of auN relative to the longest scaffold length.

Joining relatively short contigs into scaffolds with runs of N is a common practice when scaffolding assemblies. For assemblies with a high proportion of Ns, this relative auN value could be inflated by joining short contigs with long runs of N, resulting in an assembly with higher contiguity but relatively low information content compared with a similar assembly with a low proportion of Ns. To account for this aspect of quality, we also calculate a corrected auN value by substituting the square of the number of ACGT bases per scaffold into the standard auN calculation in place of scaffold length and derive a corrected relative auN value by dividing the corrected auN by the longest scaffold length, including Ns. This corrected value will be equal to the relative auN for assemblies with no Ns and below the relative auN for assemblies with a high proportion of Ns. Since this statistic has been developed in response to the visual characteristics of snail plots, we term the corrected relative auN value for an assembly the assembly “snail score.”

To investigate the relationship between snail plot appearance and snail score values, we generated a variant of the snail plot grid described above overlaid with snail score values and labeled by taxonomic kingdom. This process was automated using the docs/snail-plots/figure 6.py script in the BlobTk repository.

### Correcting for expected genome size

N50 and related values may be biased upward by overly aggressive scaffolding and filtering out too much data (Salzberg et al. 2012). Similar considerations apply to the snail score as defined above; hence, a set of corrections may be applied, depending on the available data.

As a guard against over-filtering or incomplete assembly, an expected genome size correction may be applied by providing a fixed –max-span value or specifying a –reference assembly to be parsed to extract the total assembly span. This is used to calculate a snail-G score by multiplying the snail score by the ratio of assembly span to expected size, clamped at a maximum of one.

Contrary to N50, the snail score may also be biased upward if the longest scaffolds are less than full chromosome length, leading to an increase in the uniformity of the scaffold length distribution. A basic correction for this can be applied by providing a –max-scaffold value based on a known or estimated longest chromosome length or specifying a –reference assembly from which this value can be obtained. This is used to calculate a snail-GS score by multiplying the snail-G score by the ratio of the longest scaffold length in the assembly to the expected size, again, clamped at a maximum of one.

These corrections also have a pair of absolute variants that guard against the inclusion of non-target data or uncollapsed heterozygosity (snail-aG) or overly aggressive scaffolding leading to a greater than expected longest scaffold length (snail-aGS). These absolute value corrections are calculated by taking the inverse of the respective length ratio instead of clamping if the ratio is greater than 1.

Applying these corrections depends on having a known or estimated genome size and, for the scaffold length corrections, a known or estimated value for the longest scaffold. Three of the example assemblies used above have a telomere-to-telomere (T2T) assembly available for the same species, 1 each from the 3 eukaryotic kingdoms represented. A T2T can be taken to represent the maximum achievable assembly span and longest scaffold length; hence, these assemblies provide both of the reference sizes supported by the snail plot scaling and snail score corrections.

The house mouse *Mus musculus* assembly GCA_949316315.1 (Darwin Tree of Life Project Consortium 2022) was compared with the T2T reference assembly of the C57BL/6J inbred strain, GCA_964188535.1 (Francis et al. 2025), using the command:

blobtk snail \

-d https://blobtoolkit.genomehubs.org/api/v1/dataset/id/GCA_949316315.1 \

--reference “https://ftp.ncbi.nlm.nih.gov/genomes/all/GCA/964/188/535/GCA_964188535.1_C57BL_6J_T2T_v1/GCA_964188535.1_C57BL_6J_T2T_v1_genomic.fna.gz” \

--assembly-name GCA_949316315.1 --reference-name “GCA_964188535.1 T2T”

The sacred lotus *Nelumbo nucifera* assembly GCA_003033685.1 (Gui et al. 2018) was compared with the T2T reference assembly of the China Antique cultivar, GCA_055504915.1 using the command:

blobtk snail \

-d https://blobtoolkit.genomehubs.org/api/v1/dataset/id/DLUB01.1 \

--reference “https://ftp.ncbi.nlm.nih.gov/genomes/all/GCA/055/504/915/GCA_055504915.1_China_Antique-T2T/GCA_055504915.1_China_Antique-T2T_genomic.fna.gz” \

--assembly-name GCA_003033685.1—reference-name “GCA_055504915.1 T2T”

The rice blast fungus Pyricularia oryzae assembly GCA_003016195.1 (Zhong et al. 2018) was compared with the T2T reference assembly of the P131 field strain, GCA_000292605.2 (Li et al. 2024) using the command:

blobtk snail \

-d https://blobtoolkit.genomehubs.org/api/v1/dataset/id/MQPG01

--reference “https://ftp.ncbi.nlm.nih.gov/genomes/all/GCA/000/292/605/GCA_000292605.2_PoP131/GCA_000292605.2_PoP131_genomic.fna.gz”

--assembly-name GCA_003016195.1—reference-name “GCA_000292605.2 T2T” \

All plots used the shared options --show-numbers, --show-score, and --score-type g and were exported as both SVG and YAML files to obtain a snail plot and a full set of snail score corrections.

## Results and discussion

### Snail plots visually distinguish assemblies of different qualities

Snail plots are intended to act as badges of assembly quality. The focus is on rapid interpretation of quality at a glance, but the full plots also include legends with values for a range of core statistics, including total assembly span, scaffold N50, and BUSCO completeness. This allows both visual and numerical inspection of the differences between 2 assemblies for the house mouse, *Mus musculus* (Fig. 2). Key indicators of assembly quality are more prominent in the snail plot for the chromosomal assembly GCA_949316315.1 (Figure 2a) than for the scaffold assembly GCA_000185125.1 (Figure 2b), and the detailed consideration presented in Table 1 reinforces the assertion that snail plots can reveal differences in assembly quality.

Considering the assembly composition track, it is notable that there is much less variation in mean, maximum, and minimum GC proportion along the length of the assembly for the chromosomal assembly than for the scaffold assembly. This is a feature of the plot design as base composition values are averaged across complete scaffolds; hence, a single chromosomal scaffold will contribute identical values to sets of adjacent bins. BlobToolKit supports importing windowed data for attributes such as GC proportion into a BlobDir; hence, the composition track binning could make use of these data, when available, to offer insight into patterns of base composition within chromosomes in a future version.

**Fig. 3.**
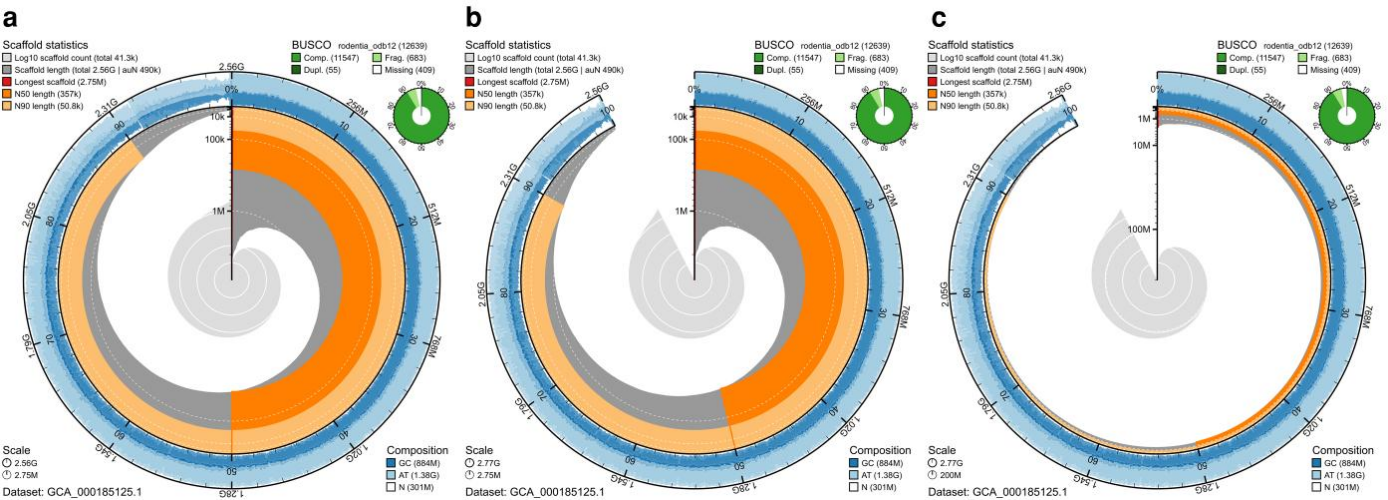
Comparison of scaling options on plots for the low-contiguity *Mus musculus* assembly GCA_000185125.1. To contrast with Fig. 2b, plots are drawn using the sqrt scaffold length scale factor instead of linear with (a) default span and scaffold scaling, b) scaled to the same total assembly span as high-contiguity assembly GCA_949316315.1, highlighting the proportional difference in span, and c) scaled to the same longest scaffold length as assembly GCA_949316315.1, emphasizing the difference in N50 and N90 lengths but producing a plot with a much lower information content.

The comparison between the 2 *Mus musculus* assemblies is based on a linear scale for scaffold lengths, which provides a more accurate representation of relative scaffold lengths than the square-root default used prior to version 0.8.0. The impact of using a square-root scale can be seen by comparing Fig. 2b with the same assembly on a square-root scale in Fig. 3a. Relative to the linear-scaled plot, the square-root scaling overemphasizes the relatively low N50 and N90 scaffold length arcs. This provided a useful distinction in the context of typically low assembly contiguity when snail plots were introduced, but could be considered misleading; hence, the default scale has been changed to linear as of BlobTk version 0.8.0. While the ability of snail plots to act as badges is dependent on consistency of scaling, this updated behavior is apparent from inspection of the scale gridlines when viewing the plot, and the change is considered justified to make the plots more accurate and useful in the era of highly contiguous assemblies.

Many of these key differences are also apparent from inspection of the set of numerical values in the plot legends that would commonly be presented in a table. The chromosomal assembly GCA_949316315.1 has longest scaffold, N50 and N90 lengths all orders of magnitude greater than the equivalent values for assembly GCA_000185125.1. There are also large differences in the number of scaffolds and percentage of missing data, with the ∼41.3k scaffolds of assembly scaffolds of assembly GCA_000185125.1 having an average 11.89% N bases compared to 0% N bases for the 179 scaffolds of assembly GCA_949316315.1. One key difference between the assemblies in Fig. 2 that is apparent from the values but not the standard snail plot presentation is that assembly GCA_949316315.1 is 210 Mb longer than assembly GCA_000185125.1. Given that these are assemblies of the same species, it is valid to rescale the plot for assembly GCA_000185125.1 to match the greater assembly span of the chromosomal assembly (Fig. 3b), highlighting the proportional difference between the total span of the 2 assemblies. The radial axis may also be rescaled to match (Fig. 3c); however, with such a large difference in longest scaffold length, this generates a plot with mostly white space. While this serves to highlight the difference in assembly quality, it does so at the expense of providing insight into the distribution of scaffold lengths in the less contiguous assembly.

Supporting the use of snail plots as a visual representation of assembly quality, we have introduced an option to generate the plots with no text or labels as a true assembly badge. Here, consistent scaling is more important than in the standard plot as the visual cues to the differences between a square-root and linear scale function may not be visible when the badge is very small. To ensure fair comparison between snail plot assembly badges, the scale function is fixed to linear when the badge option is used.

### Snail plots allow deeper insight than individual metrics

**Fig. 4.**
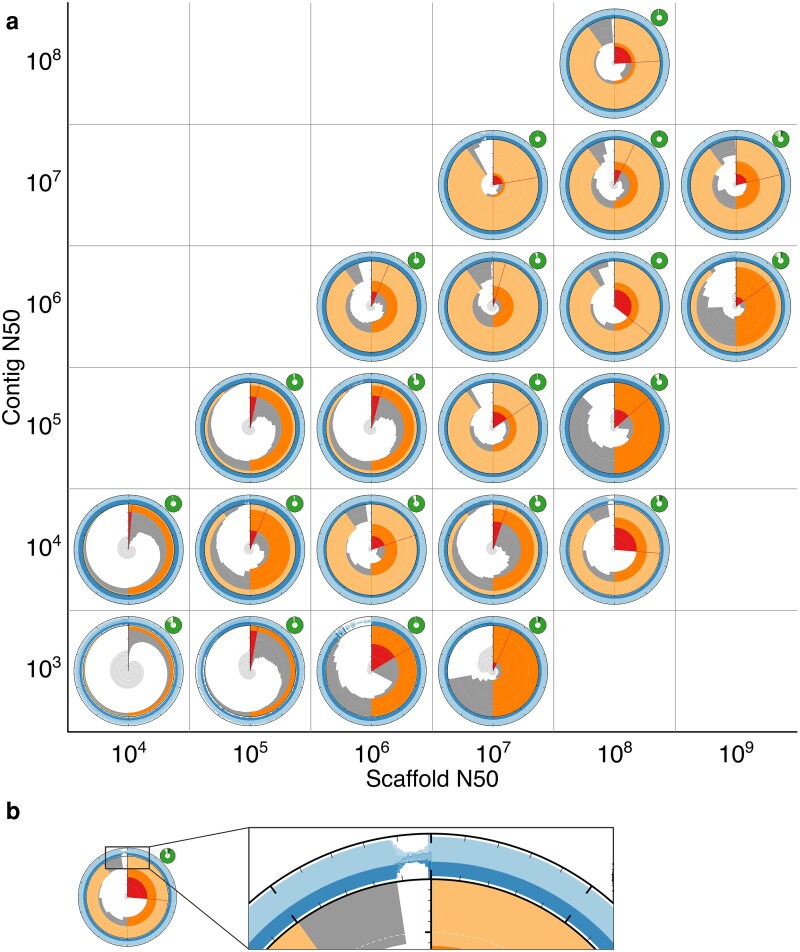
A set of linear-scaled snail plots for assemblies of varying quality arranged by order of magnitude of assembly contig and scaffold N50 values. Plots are presented as assembly badges to highlight the suitability of the plots for qualitative assessment of assemblies. a) While features such as the amount of data ink (proportional to auN statistics) are broadly correlated with scaffold and contig N50, there are a number of differences that are less closely correlated with variation along these axes. b) Enlarged section of *Nelumbo nucifera* assembly GCA_003033685.1 (contig N50 = 10^4^, scaffold N50 = 10^8^) to show the presence of whitespace, indicating Ns in the longest and shortest scaffolds.

Arranging a set of snail plot assembly badges for assemblies of varying quality along axes of contig and scaffold N50 (Fig. 4) reveals some correlation with indicators of assembly quality but highlights further assembly features that are not directly related to these primary axes. Details of the assemblies included in this plot are shown in Table 3. Individual assemblies in this figure are referred to using the Vertebrate Genome Project convention of referring to an assembly by the order-of-magnitude of the Contig and scaffold N50 lengths, respectively (Rhie et al. 2021), e.g. the last assembly on the bottom row of the plot with a 10^3^ contig N50 and a 10^7^ scaffold N50 would be referred to as the assembly at 3.7.

**Table 3.** Selected assembly statistics for assemblies included in the assessment of correlation between N50 values and snail plot assembly badge features.

Accession	Scientific name	Assembly level	Assembly span (Mbp)	Contig N50 bin	Scaffold N50 bin	Longest scaffold (Mbp)	N portion	auN (Mbp)	Relative auN	Adjusted auN (Mbp)	Snail score	BlobToolKit ID	Ref
GCA_019009955.1	*Riptortus pedestris*	Chromosome	1,079.52	8	8	260.32	0.00	185.42	0.71	185.42	0.71	JADPXZ01	Huang et al. 2021
GCA_000001215.4	*Drosophila melanogaster*	Chromosome	143.71	7	7	32.08	0.01	24.92	0.78	24.82	0.77	GCA_000001215_4	dos Santos et al. 2015
GCA_949316315.1	*Mus musculus*	Chromosome	2,770.95	7	8	200.13	0.00	136.65	0.68	136.64	0.68	GCA_949316315.1	Darwin Tree of Life Project Consortium 2022
GCA_963693085.1	*Alisma plantago-aquatica*	Chromosome	9,377.97	7	9	1,999.79	0.00	1,454.72	0.73	1,454.70	0.73	GCA_963693085.1	Christenhusz et al. 2025
GCA_964340765.1	*Umbilicaria deusta*	Chromosome	40.76	6	6	2.54	0.00	1.63	0.64	1.63	0.64	GCA_964340765.1	Darwin Tree of Life Project Consortium 2022
GCA_964340405.1	*Actinotia polyodon*	Scaffold	626.61	6	7	27.89	0.00	21.63	0.78	21.63	0.78	GCA_964340405.1	Darwin Tree of Life Project Consortium 2022
GCA_963691655.1	*Toxonevra muliebris*	Chromosome	491.42	6	8	175.30	0.00	118.95	0.68	118.92	0.68	GCA_963691655.1	Barclay et al. 2025
GCA_964205295.1	*Bombina variegata*	Chromosome	9,369.77	6	9	1,415.94	0.00	937.76	0.66	937.48	0.66	GCA_964205295.1	—
GCA_002222395.1	*Cryptococcus neoformans var. grubii* MW-RSA36	Scaffold	18.45	5	5	0.62	0.00	0.21	0.34	0.21	0.34	AMLD01	—
GCA_000204055.1	*Melampsora larici-populina* 98AG31	Scaffold	101.13	5	6	4.07	0.03	1.41	0.35	1.34	0.33	AECX01.1	Duplessis et al. 2011
GCA_018257905.1	*Gillenia trifoliata*	Chromosome	296.28	5	7	46.31	0.00	29.15	0.63	29.01	0.63	JAEHOF01	Ireland et al. 2021
GCA_013339765.2	*Haemaphysalis longicornis*	Chromosome	2,554.73	5	8	348.26	0.00	198.69	0.57	198.61	0.57	JABSTR01.1	Jia et al. 2020
GCA_003016195.1	*Pyricularia oryzae*	Scaffold	37.97	4	4	0.53	0.00	0.13	0.24	0.13	0.24	MQPG01	Zhong et al. 2018
GCA_000261425.2	*Cladosporium sphaerospermum* UM 843	Scaffold	26.89	4	5	1.69	0.01	0.86	0.51	0.85	0.51	AIIA02	Ng et al. 2012
GCA_001661245.1	*Pachysolen tannophilus* NRRL Y-2460	Scaffold	12.60	4	6	2.51	0.02	1.69	0.67	1.62	0.65	LZCH01	Riley et al. 2016
GCA_014337955.1	*Aspidoscelis marmoratus*	Scaffold	1,639.53	4	7	85.03	0.06	40.27	0.47	36.03	0.42	MTQE01	—
GCA_003033685.1	*Nelumbo nucifera*	Chromosome	817.27	4	8	215.18	0.13	119.46	0.56	91.26	0.42	DLUB01.1	Gui et al. 2018
GCA_900322205.1	*Kewa caespitosa*	Scaffold	664.63	3	4	0.34	0.19	0.04	0.12	0.03	0.08	ONZA01	—
GCA_001632505.1	*Daphnia magna*	Scaffold	129.54	3	5	3.72	0.18	0.85	0.23	0.58	0.16	LRGB01	—
GCA_020883555.1	*Drosophila nannoptera*	Scaffold	134.50	3	6	22.04	0.15	11.21	0.51	9.44	0.43	JABVZY01	—
GCA_013467465.1	*Gossypium trilobum*	Chromosome	655.38	3	7	44.53	0.04	26.81	0.60	23.72	0.53	JABEZW01	—

Considering the key features highlighted above, assemblies toward the top right of the plot appear to have higher quality than those at the bottom left, sharing prominent N50 and N90 overlays, a stepped scaffold length curve that fills much of the plot area, and a small scaffold count curve. By contrast, assemblies toward the bottom left have prominent scaffold count curves, smooth scaffold length curves, and less prominent N50 and, in particular, N90 overlays. Among assemblies with relatively high N90, there is a distinction between those for which scaffold lengths in each bin are within an order of magnitude of the longest scaffold length and those with a large number of relatively short scaffolds showing as a gap in the circularized scaffold length distribution. For *Drosophila melanogaster* assembly GCA_000001215.4 at 7.7 (contig N50 = 10^7^, scaffold N50 = 10^7^), this is accompanied by an increase in sequence composition variability and/or proportion of Ns.

Some assemblies with low contig N50 (in the 10^3^ and 10^4^ bp bins) have scaffold N50 several orders of magnitude greater than contig N50. These typically show signatures of low-quality assemblies as discussed above, but the assemblies at 4.6 (contig N50 = 10^4^, scaffold N50 = 10^6^) and 4.8 (contig N50 = 10^4^, scaffold N50 = 10^8^) (*Pachysolen tannophilus* NRRL Y-2460 assembly GCA_001661245.1 and *Nelumbo nucifera* assembly GCA_003033685.1, respectively) have several signatures of high-quality assemblies. A key difference between these assemblies is that assembly GCA_001661245.1 at 4.6 has an almost completely shaded sequence composition track, reflecting an overall proportion of Ns < 2%, which assembly GCA_003033685.1 at 4.8 has whitespace at the edges all around this track, reflecting >13% Ns across the whole assembly (Fig. 4b). Assembly GCA_001661245.1 could therefore be judged to be of higher overall quality.

### Snail plots support comparisons across taxa

**Fig. 5.**
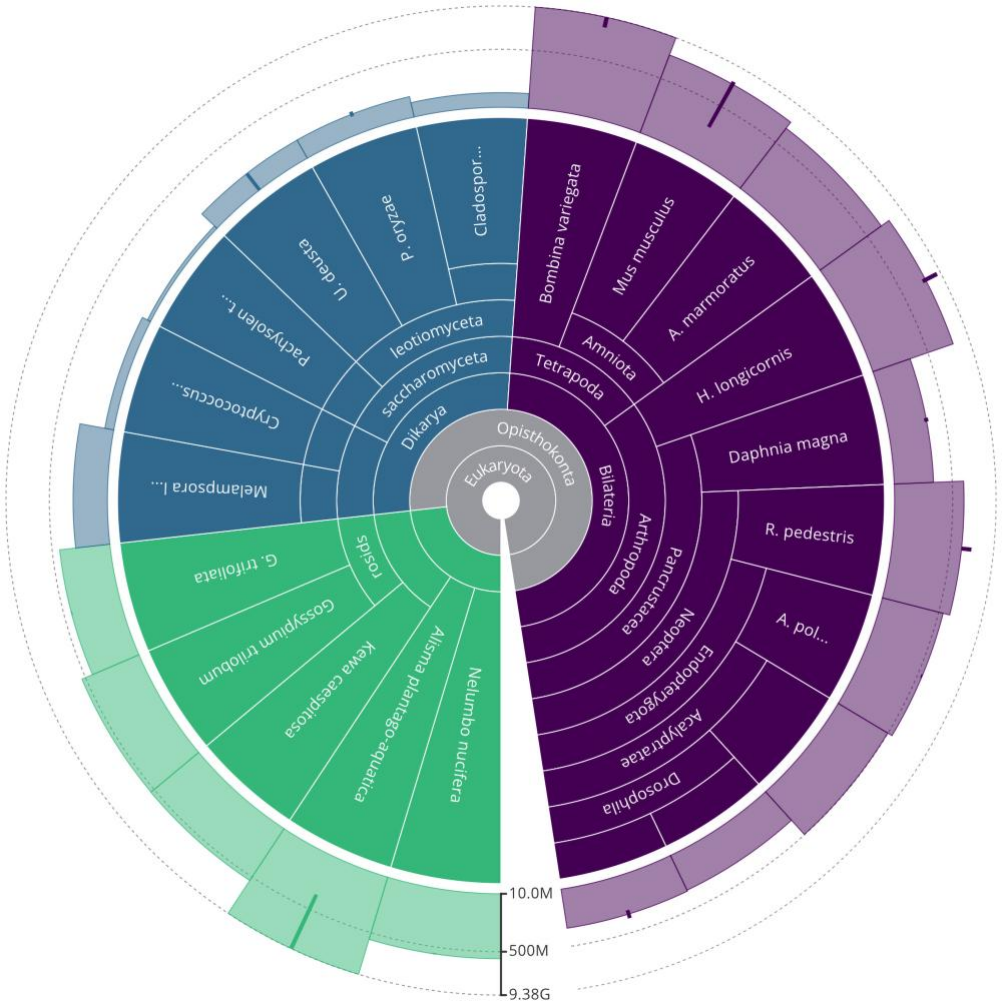
Taxonomic distribution and sizes of assemblies included in the assessment of correlation between N50 values and snail plot assembly badge features. Taxa are colored by kingdom to highlight plant (green), fungus (blue), and animal (purple) genomes. Bars show the span of each assembly ranging from 12.6Mb to 9.38Gb. Report generated using Genomes on a Tree.

In comparative genomics, the power of an analysis is typically limited by the lowest quality assembly, creating a need for tools and resources to compare quality across taxa (Feron and Waterhouse 2022). While BUSCO scores can be directly compared across taxa, many other metrics are inherently correlated with the genome or assembly size of each taxon. This limitation is explicitly acknowledged by some large-scale biodiversity sequencing initiatives such as the Earth BioGenome Project, which has minimum contig NG50 criteria for assemblies that are relaxed for assemblies where the chromosomal NG50 would be too low to meet the threshold (Lawniczak et al. 2022). Placing the assemblies selected for the comparison above on a taxonomy tree using Genomes on a Tree (GoaT) shows that the assemblies span the tree of life and have a range of assembly spans from 12.6 Mb to 9.4 Gb (Fig. 5).

This demonstrates the effectiveness of snail plots for providing insight into markers of assembly quality across 3 kingdoms in the Eukaryota and across almost 4 orders of magnitude. This result emphasizes the scale independence of the plots and suggests that they should have utility in selecting assemblies for inclusion in comparative analyses. While there are biological constraints that should be considered when comparing plots across taxa with widely diverging genome architecture, features such as high vs low chromosome count and asymmetric or bimodal karyotypes should have recognizable signatures in a snail plot, potentially allowing reasons for apparent quality differences to be inferred.

### Snail plots suggest a new assembly quality metric

The interpretation of quality above emphasizes the importance of the area covered by the scaffold length curve, including the associated overlays. This is closely related to auN; however, auN lacks the scale independence of snail plots since the maximum possible value of auN increases with assembly span. To correct for this, we propose using a relative auN score by dividing auN by the longest scaffold length to obtain a score between 0 and 1. This relative auN score reflects the way that the visual presentation of a snail plot is scaled to the length of the longest scaffold when drawing snail plots and scores for the assemblies in Fig. 4 are shown in Table 3.

**Fig. 6.**
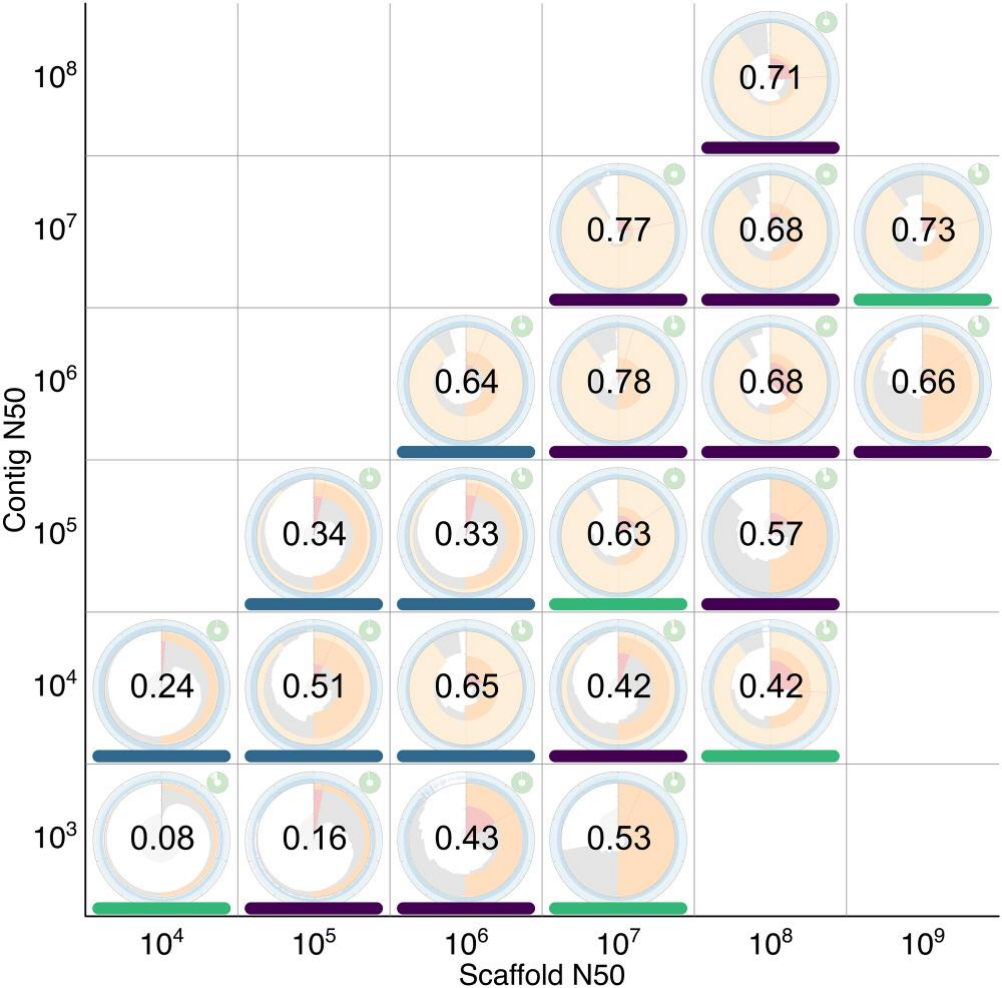
Snail scores (relative measure of auN adjusted for ambiguous bases) for the assemblies in Fig. 4. Colored bars indicate the taxonomic kingdom, plant (green), fungus (blue), and animal (purple), for each assembly.

To account for the proportion of ambiguous bases (Ns), another key indicator of quality, an adjusted variant of the relative auN score can be used. The adjustment is based on squaring the count of ACGT bases in each scaffold when calculating auN instead of simply squaring the scaffold lengths. There is a good correlation between snail plot features associated with higher assembly quality and this corrected value, which we term the assembly “snail score.” While this is necessarily a somewhat subjective correlation, as shown in Fig. 6, snail scores broadly increase with contig and scaffold N50. The highest snail scores of 0.77 and 0.78 are obtained by assemblies at 7.7 (contig N50 = 10^7^, scaffold N50 = 10^7^) and 6.7 (contig N50 = 10^6^, scaffold N50 = 10^7^), *D. melanogaster* assembly GCA_000001215.4, and *Actinotia polyodon* assembly GCA_964340405.1, respectively. Among the assemblies with contig N50 values below 10^6^, the 2 assemblies with the most visual indicators of high assembly quality are the only assemblies with snail scores above 0.6, these are *Gillenia trifoliata* assembly GCA_018257905.1 at 5.7 (contig N50 = 10^5^, scaffold N50 = 10^7^) with a score of 0.63 and *Pachysolen tannophilus* NRRL Y-2460 assembly GCA_001661245.1 at 4.6 (contig N50 = 10^4^, scaffold N50 = 10^6^) with a score of 0.65. *Nelumbo nucifera* assembly GCA_003033685.1 at 4.8 (contig N50 = 10^4^, scaffold N50 = 10^8^), which was contrasted with GCA_001661245.1 above as having visual markers of high assembly quality, but a high proportion of Ns achieves an unadjusted (relative auN) score of 0.56, but an adjusted, snail score of 0.42.

Based on this sample of assemblies, it is apparent that snail scores correlate with visual markers of assembly quality, and while acknowledging that any cutoff is necessarily arbitrary, we suggest that a score above ∼0.6 can be taken as a broad indication of assembly quality. Assemblies within the sample set that achieved a score above this cutoff were drawn from 3 kingdoms of Eukaryota and had a range of assembly spans from 12.6 Mb to 9.4 Gb. Thus, snail scores provide a single value indicator of assembly quality, with higher values associated with more markers of assembly quality, allowing them to be used for assessments of relative assembly quality across a wide range of taxonomic and assembly size diversity. It should be noted, however, that the maximum possible snail score for a taxon is limited by the relative physical sizes of its chromosomes. Taxa with a single large chromosome and a number of much smaller chromosomes will have relatively low snail scores, even for a full telomere-to-telomere (T2T) assembly.

### Snail plots support comparison to a reference assembly

**Fig. 7.**
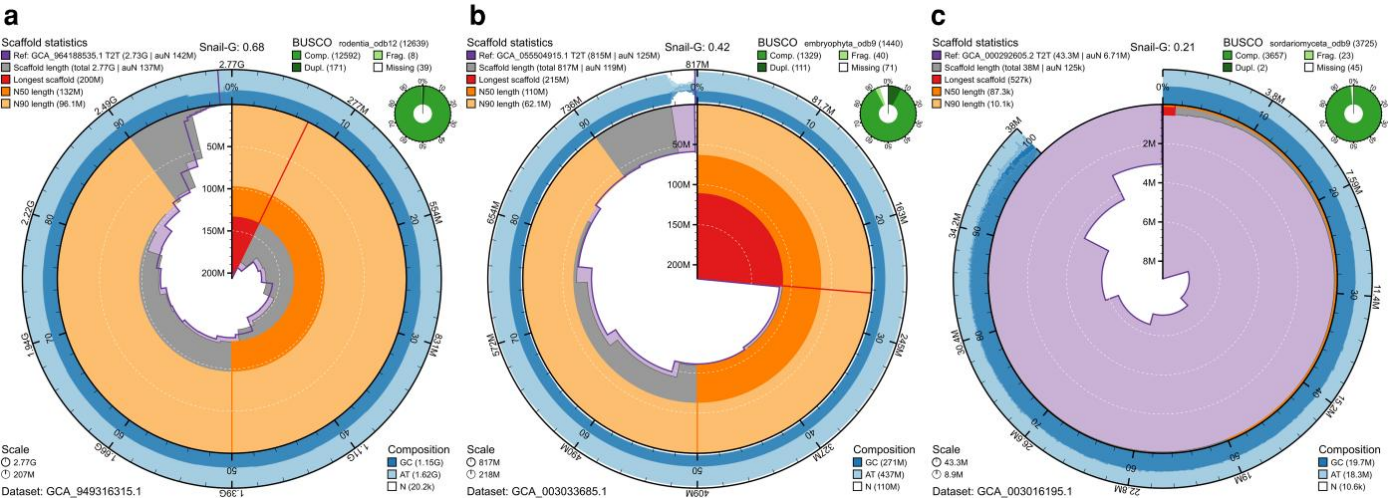
Comparison of selected assemblies used in this study with telomere-to-telomere (T2T) reference assemblies. Purple lines and shading in each snail plot represent the scaffold length distributions of the corresponding T2T assembly. a) House mouse, *Mus musculus*, assembly GCA_949316315.1 compared with T2T assembly GCA_964188535.1. b) Sacred lotus, *Nelumbo nucifera*, assembly GCA_003033685.1 compared with T2T assembly GCA_055504915.1. c) Rice blast fungus, *Pyricularia oryzae*, assembly GCA_055504915.1 compared with T2T assembly GCA_000292605.2.

For 3 of the assemblies in this comparison, full T2T assemblies are available so we compared the *Mus musculus* (GCA_949316315.1), *Nelumbo nucifera* (GCA_003033685.1) and *Pyricularia oryzae* (GCA_003016195.1) assemblies with their respective T2T assemblies: *Mus musculus* C57BL/6J inbred strain assembly GCA_964188535.1, *Nelumbo nucifera* China Antique cultivar assembly GCA_055504915.1, and *Pyricularia oryzae* P131 field strain assembly GCA_000292605.2. The resulting reference-scaled plots (Fig. 7) show that both the *Mus musculus* and *Nelumbo nucifera* assemblies share similar scaffold length distributions with their respective T2T reference assemblies, with the *Mus musculus* assembly having a mix of longer and shorter size-sorted scaffolds and greater overall span that the reference (Fig. 7a), while *Nelumbo Nucifera* had typically shorter scaffold lengths than the reference beyond N50 (Fig. 7b), noting as previously mentioned the high proportion of Ns in the apparently highly contiguous sequence. In contrast, the scaffold length distribution curve for *Pyricularia oryzae* is barely visible around the edge of the reference-scaled plot, reflecting much lower contiguity and significantly shorter overall assembly span than the reference (Fig. 7c).

The reference assemblies were also used to calculate corrected snail scores for the 3 target assemblies (Table 4). Each of the T2T reference assemblies had a higher base snail score than the corresponding target assembly. In line with the observations from comparing the snail plots, the difference was smallest for *Mus musculus* and greatest for *Pyricularia oryzae*. The high number of ambiguous bases (Ns) in the *Nelumbo Nucifera* is reflected in the relatively large snail score difference between the target (0.424) and reference (0.573) assemblies, despite the broadly congruous scaffold size distributions, emphasizing the impact of the correction for ambiguous bases in the snail score calculation.

**Table 4.** Reference-adjusted snail scores for the assemblies presented in Fig. 7.

Species	Target assembly			Reference assembly				Target assembly snail scores				
	Accession	Span (Mb)	Scaf (Mb)	Accession	Span (Mb)	Scaf (Mb)	Snail Score	Base	G	GS	aG	aGS
*Mus musculus*	GCA_949316315.1	2,771.0	200.1	GCA_964188535.1	2,731.3	206.8	0.686	0.683	0.683	0.661	0.673	0.661
*Nelumbo nucifera*	GCA_003033685.1	817.3	215.2	GCA_055504915.1	815.2	217.5	0.573	0.424	0.424	0.419	0.423	0.419
*Pyricularia oryzae*	GCA_055504915.1	38.0	0.5	GCA_000292605.2	43.3	8.9	0.754	0.237	0.208	0.012	0.208	0.012

Applying an expected genome size correction (snail-G score) to the *Mus musculus* and *Nelumbo nucifera* snail scores had no effect, as both have a total assembly span greater than the corresponding T2T reference. The absolute correction gave a slightly reduced snail-aG score in each case, from 0.683 to 0.661 for *Mus musculus* and from 0.424 to 0.419 for *Nelumbo nucifera*. For *Pyricularia oryzae* with a shorter assembly span than the reference, the snail-G score was lower than the base score (reduced from 0.237 to 0.208).

For all assemblies, the longest scaffold was shorter than for the T2T reference; hence, all showed a reduced snail-GS score relative to the base snail score. Corresponding to the differences observed in the plots, this difference was greatest for *Pyricularia oryzae,* for which the snail-GS score is only 0.012.

Collectively, these corrections should allow robust score comparisons that guard against score inflation through misassembly or trivial assembly manipulations such as overly aggressive scaffolding and filtering out too much data that may affect uncorrected metrics (Salzberg et al. 2012). However, it should be noted that the snail scores for the T2T assemblies themselves range from 0.573 to 0.754; hence, comparisons across taxa with divergent genome architecture must be made with caution, and users should refer to the corresponding plots to identify signatures such as the apparently asymmetric karyotype of *Nelumbo nucifera*. This species has the T2T assembly with the lowest snail score (0.573), and the karyotypic asymmetry is evident from the red segment representing the longest scaffold occupying over one-quarter of the plot and the large step down to the next longest scaffold length.

Software such as QUAST (Gurevich et al. 2013) makes much greater use of reference assemblies when available through statistics and distributions inferred from an alignment of the target and reference assemblies. While it is not practical to apply this approach to snail plots, which are constructed from the BlobToolKit-derived model of independently analyzed datasets for each assembly, it would be possible to assign, split, and order scaffolds in an assembly based on alignment to a reference prior to constructing a snail plot. Breaking misassembled scaffolds, in particular, would lead to a reduction in the area of the scaffold length curve and any affected overlays, which would be reflected in the snail score. A future version could add support for using a fixed scaffold order to present a reference-aligned snail plot.

## Conclusions

Snail plots have become a widely used representation of core genome assembly metrics that facilitate rapid identification of characteristic markers of both high and low assembly quality. These markers of quality are broadly correlated with assembly N50 values, and perhaps most closely with metrics that consider the full distribution of N*x* values. Unlike this family of metrics, snail plots present this information in a scale-independent way, similar to plots of the full N*x* curve. Based on this observation, we have suggested modifying the area under N (auN) calculation to account for ambiguous bases and scale to a proportion of the maximum auN score possible, given the assembly span and the longest scaffold length. The resulting snail score correlates closely with a visual assessment of assembly quality markers in a set of snail plots and supports adjustments for expected genome size analogous to NG50 and auNG metrics. While snail scores could fulfil the requirement for a single tabulatable value to indicate assembly quality across a range of genome sizes, interpretation of the scores must take into account differences in genome architecture. As such, the more holistic view of assembly quality afforded by the full snail plot provides a much richer insight, which can accommodate and facilitate the identification of genomic features that may bias the snail score.

## Data Availability

The blobtk plot and blobtk snail commands are available under an MIT license from the BlobTK GitHub repository at https://github.com/genomehubs/blobtk. This repository also contains scripts and commands used to generate figures presented in this study under the docs/snail-plots directory. Version 0.8.0 used in this study is archived at https://zenodo.org/records/19005725. All data analyzed are publicly available via https://goat.genomehubs.org/2025.04.21/and from https://blobtoolkit.genomehubs.org.
